# Oxidative stress preconditioning of mouse perivascular myogenic progenitors selects a subpopulation of cells with a distinct survival advantage in vitro and in vivo

**DOI:** 10.1038/s41419-017-0012-9

**Published:** 2018-01-03

**Authors:** Cesare Gargioli, Giuseppina Turturici, Maria M. Barreca, Walter Spinello, Claudia Fuoco, Stefano Testa, Salvatore Feo, Stefano M. Cannata, Giulio Cossu, Gabriella Sconzo, Fabiana Geraci

**Affiliations:** 1 Department of Biology, University of Rome “Tor Vergata”, Rome, 00133 Italy; 2 Department of Biological, Chemical and Pharmaceutical Sciences and Technologies, University of Palermo, Palermo, 90128 Italy; 3 Division of Cell Matrix Biology and Regenerative Medicine, University of Manchester, Oxford Road, M13 9PL Manchester, UK; 4 Euro-Mediterranean Institute of Science and Technology, Palermo, Italy

## Abstract

Cell engraftment, survival and integration during transplantation procedures represent the crux of cell-based therapies. Thus, there have been many studies focused on improving cell viability upon implantation. We used severe oxidative stress to select for a mouse mesoangioblast subpopulation in vitro and found that this subpopulation retained self-renewal and myogenic differentiation capacities while notably enhancing cell survival, proliferation and migration relative to unselected cells. Additionally, this subpopulation of cells presented different resistance and recovery properties upon oxidative stress treatment, demonstrating select advantages over parental mesoangioblasts in our experimental analysis. Specifically, the cells were resistant to oxidative environments, demonstrating survival, continuous self-renewal and improved migration capability. The primary outcome of the selected cells was determined in in vivo experiments in which immunocompromised dystrophic mice were injected intramuscularly in the tibialis anterior with selected or non-selected mesoangioblasts. Resistant mesoangioblasts exhibited markedly enhanced survival and integration into the host skeletal muscle, accounting for a more than 70% increase in engraftment compared with that of the unselected mesoangioblast cell population and leading to remarkable muscle recovery. Thus, the positive effects of sorting on mesoangioblast cell behaviour in vitro and in vivo suggest that a selection step involving oxidative stress preconditioning may provide a novel methodology to select for resistant cells for use in regenerative tissue applications to prevent high mortality rates upon transplantation.

## Introduction

The release of several types of factors, such as cytokines and growth factors, from damaged tissues stimulates both resident and circulating stem cells to initiate tissue repair programmes.^1–3^ However, the therapeutic efficacy of stem cells is compromised by reduced homing towards the target site^4, 5^ and by the cytotoxic environment, which causes massive cell death during the first several days post-transplantation.^5–9^ For this reason, enhancing in vivo stem cell viability may be a key step in improving the outcomes of cell-based therapies. The microenvironment within damaged tissues is unfavourable for stem cell survival due to hypoxia, inflammatory mediators, a lack of glucose or serum and oxidative stress, with the latter being particularly detrimental.^6,10,11^ In particular, hydrogen peroxide (H_2_O_2_), a reactive oxygen species (ROS) that diffuses freely into and out of cells,^12,13^ may play a significant role in inducing the apoptosis or necrosis of injected stem cells.^13–15^ Although the regulation of cell death by external oxidative stress has been extensively studied in vitro, these experiments typically use differentiated cells rather than stem cells and focus on events that occur shortly after treatment (i.e., a few minutes later or at most in the first 24 h).^16,17^ In the field of stem cell research, in vitro experiments based on comparative analyses of oxidative stress resistance among mesenchymal stem cells, embryonic stem cells and induced pluripotent stem cells (iPSCs) have shown that iPSCs and embryonic stem cells are less resistant to oxidative stress than mesenchymal stem cells.^18^ However, other studies have demonstrated that oxidative stress induces senescence in human mesenchymal stem cells.^19–21^ Therefore, despite its central role in the development of cell-based therapies, the effects of exogenous oxidative stress on stem cell viability are not well understood. To explore the reasons why only a few stem cells survive after transplantation, we first performed an in vitro study. H_2_O_2_ was used to apply extreme exogenous oxidative stress to mouse mesoangioblast perivascular myogenic progenitors (hereafter referred to as ‘mabs’ or ‘A6 cells’) to isolate resistant cells that survived after a long recovery period. The resistant cells (hereafter ‘cell clones’ or ‘H2 cells’) exhibited the unusual ability to retain self-renewal capacity in addition to increased migratory and proliferation capabilities with respect to the untreated mab population. Moreover, in vivo experiments involving the intramuscular injection of cell clones into immunocompromised dystrophic mice further highlighted noteworthy improvements in cell survival, migration and engraftment into host skeletal muscle tissue compared with those of unstressed cells. Mabs are easily expandable in vitro and have largely been studied for cell-based therapeutic applications; thus, they are prime candidates for skeletal muscle regeneration and reconstruction.^22–27^ Therefore, mabs derived from the selected subpopulation are better able to tolerate oxidative stress and display distinct survival and integration advantages in vivo upon transplantation, representing an important approach to potentiate improvements in mab-based cell therapy.

## Results

### Different H_2_O_2_ doses and exposure times affect mab cell cycle progression and viability

**Fig. 1:**
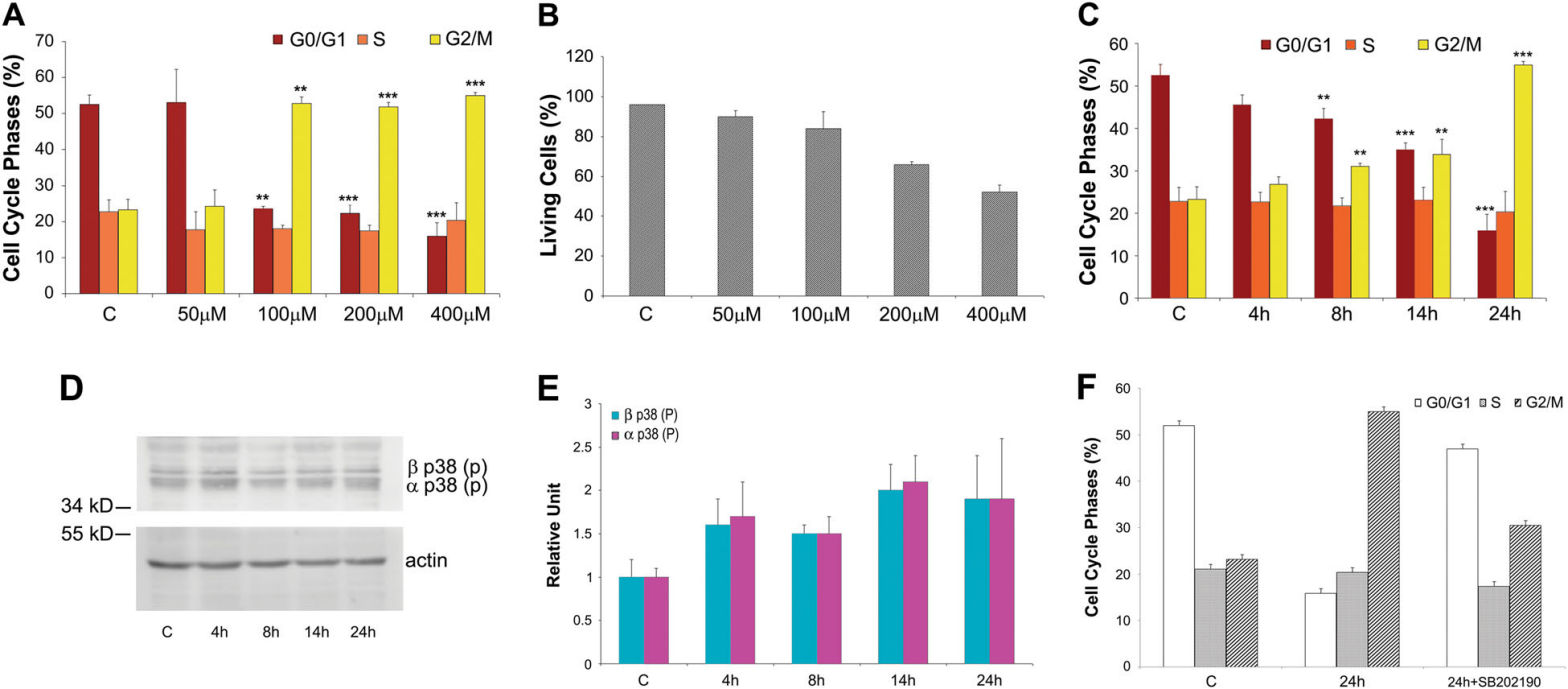
Selection of growth culture condition for experiments on A6 cells **a** Histograms  of cell cycle phase distribution: control cells 'C' and treated cells for 24 h with H_2_O_2_: 50, 100, 200, and 400 μM. **b** Effects of oxidative stress on cell viability: control cells 'C' and treated cells for 24 h with 50, 100, 200, and 400 μM. H_2_O_2_ assayed by trypan blue exclusion assays. **c** Histograms of cell cycle phase distribution during 24 h of treatment: control cells 'C' and treated cells with 400 μM H_2_O_2_ for 4, 8, 14 and 24 h. All data in histograms A, B and C were obtained from three independent experiments. The error bars indicate standard deviation. **P < 0.05, ***P < 0.005 vs control group. p38 is responsible for cell cycle progression. **d-e** Phosphorylated p38 kinase level of A6 cells after a 400 μM H_2_O_2_ 24 h treatment. Western blot **d** and quantification **e** of the two phosphorylated p38 kinase isoforms. Phosphorylated p38 levels in control A6 cells 'C' and in treated A6 cells (400 μM H_2_O_2_ for 4, 8, 14, 24 h). The levels of p38 were valuated after quantification of immunoreactive bands by Quantity One software. Data were obtained from three independent experiments. The error bars indicate standard deviation. **f** Histograms of cell  cycle phase distribution of treated A6 cells in presence of the p38 inhibitor SB202190. Control A6 cells 'C', treated A6 cells (400 μM H_2_O_2_ for 24 h) '24 h', treated (as 24 h) A6 cells with p38 inhibitor '24 h + SB202190'. Displayed are typical histograms from three independent experiments. Error bars indicate standard deviation

To select resistant cells that survive in an oxidative environment, we identified a sub-lethal concentration of H_2_O_2_ that inhibited cell cycle progression and partially killed the treated cells. We analysed the dose responses of sub-confluent mab cultures treated with varying doses of H_2_O_2_ and determined that treatment with 400 μM H_2_O_2_ for 24 h resulted in cell cycle arrest in the G_2_/M phase (Fig. 1a) and 50% cell survival (Fig. 1b), representing optimal conditions to isolate oxidative stress-resistant mabs. Cell cycle analysis by cytofluorimeter revealed higher G_2_/M phase arrest after exposure for 24 h of but not at shorter time points (i.e., 4, 8, or 14 h), indicating that arrest was strictly dependent on the length of exposure (Fig. 1c). This high dose was the maximum theoretical H_2_O_2_ concentration achievable in tissues with severe inflammation. Thus, the highest tolerable concentration that blocks mitosis and does not immediately induce complete death in mabs was chosen to select for the oxidative stress-resistant subpopulation.

We also investigated the p38 activation pathway, which may participate in cell cycle arrest during mitosis (G_2_/M phase) due to Cdc25B inhibition resulting from increased phosphorylated p38 MAPK activity.^28,29^ To explore whether this same mechanism occurs in mabs upon oxidative stress exposure (400 μM H_2_O_2_), we evaluated p38 MAPK levels after H_2_O_2_ exposure for different periods of time (4, 8, 14 and 24 h) when we observed mitotic blockade (see Fig. 1c). Western blotting (Figs. 1d, e) showed that both alpha- and beta-phosphorylated isoform levels increased during oxidative stress at these same time points when we observed mitosis blockade. This association appeared functionally relevant because mabs treated with both 400 μM H_2_O_2_ and a p38 MAPK inhibitor (SB202190) did not exhibit G_2_/M phase blockade (Fig. 1f). This finding supports a role for p38 MAPK activity in cell cycle blockade due to oxidative stress.

### Cell viability, proliferation and ROS generation during the recovery period

**Fig. 2:**
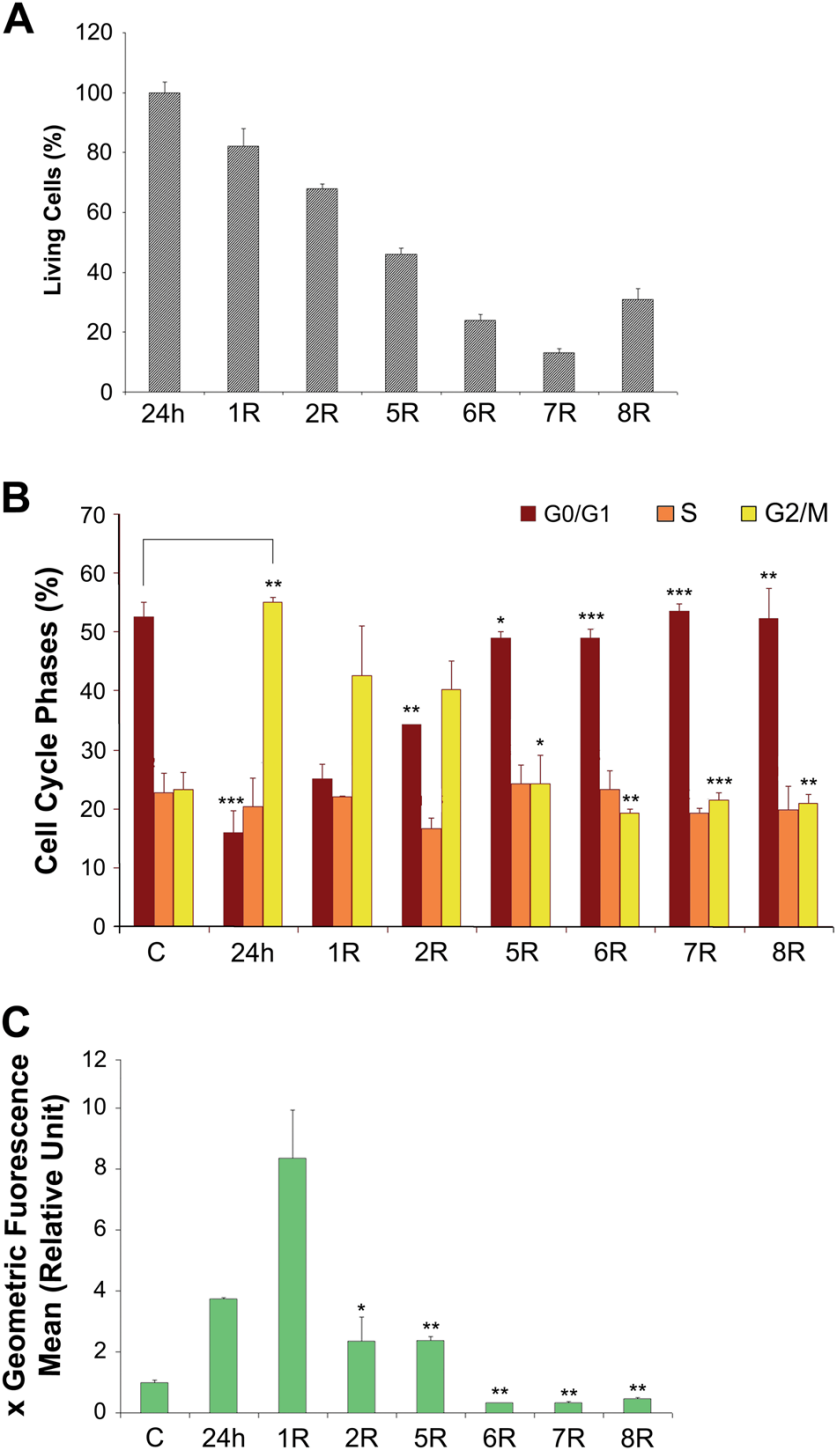
Effect of 400 μM H_2_O_2_ treatment on A6 cells during recovery time **a** Histograms of cell viability assayed by trypan blue exclusion assays: treated cells '24 h' with 400 μM H_2_O_2_ and after 1, 2, 5, 6, 7 and 8 days of recovery (R) calculated respect to 24 h. **b** Histograms of cell cycle phase distribution: control cells 'C', treated cells (400 μM H_2_O_2_ 24 h) and after 1, 2, 5 and 8 days of recovery are represented. **c** Histograms of ROS content: control cells “C”, treated cells (400 μM H_2_O_2_ 24 h) and after 1, 2, 5, 6, 7 and 8 days of recovery are represented. The ROS values of samples were reported in graph as multiple of cell control value (see methods). All data were obtained from three independent experiments. Error bars indicate standard deviation. *P < 0.5, **P < 0.05, ***P < 0.005 vs control group, or H_2_O_2_ group

To study long-term survival in H_2_O_2_-treated mabs, specific analyses were performed during the recovery period. Throughout recovery, a portion of the treated cells died, as assessed by trypan blue exclusion assays. This trend began to reverse on the eighth day of recovery (Fig. 2a). Simultaneously, during the 1 days of recovery, the cells were still blocked in G_2_/M phase (Fig. 2b); cell cycle distribution returned to normal on day five. Thus, although the cell cycle was restored, cell death continued daily. The intracellular ROS concentration was maximal on the 1 day of recovery, indicating that at the end of treatment, the stressed cells were producing large quantities of endogenous ROS (Fig. 2c).

### H_2_O_2_ mab treatment facilitates the isolation of resistant cell clones

On day 8, proliferation started to prevail over cell death. Treated cells after day eight of recovery were plated at clonal dilutions and subsequently grown for an additional 15 days to sort resistant clones. The plated cells continued to die during the subsequent 15 days of recovery, and only a few cells survived to form clones. The surviving cell clones were then subjected to a second round of oxidative stress by applying the same sub-lethal treatment (400 μM H_2_O_2_ for 24 h). Based on a cell cycle analysis of three clones (H2, E3, B9), lower percentages of cells were blocked in G_2_/M phase after treatment than that of mabs (Supplementary Figs. S1A-D). The three cell clones were similar, and for the remainder of the study, we carefully analysed the H2 clone to determine whether it preserved the mab phenotype while possessing enhanced resistance to oxidative stress.

### The H2 clone retains the basal mab phenotype

**Fig. 3:**
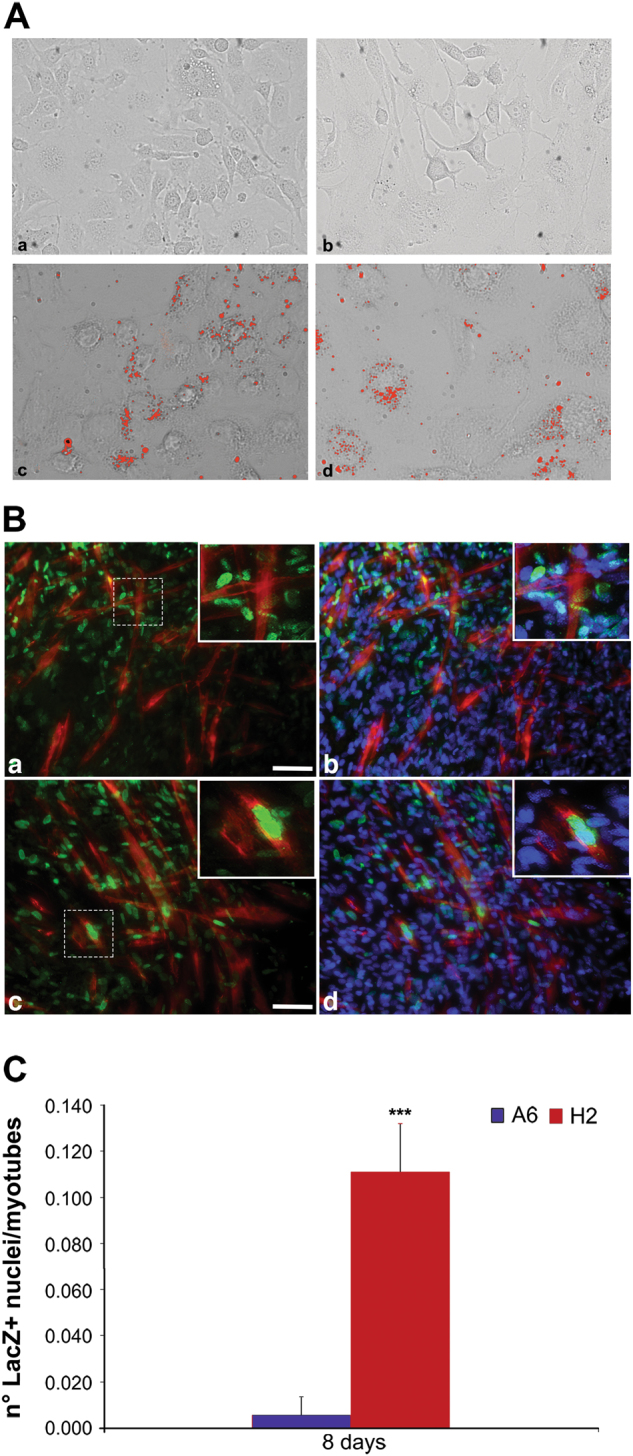
Differentiation of A6 cells and H2 cell clone in vitro **a** A6 cells **a** and cell clone **b** after differentiation in adipocytes, A6 cells **c** and cell clone **d** differentiated in adipocyte and stained by oil red. Showed are typical photographs with 20X magnification. Scale bar is 15 μm. All data were obtained from three independent experiments. **b** A6 cells **a, b** and cell clone **c, d** labeled with lentiviral infection with nuclear nLacZ differentiated in myotube in co-culture with C2C12 mouse myoblast cell line. Immunofluorescence against LacZ (green) and Myosin Heavy Chain (MyHC) (red) revealing H2 nuclei participation in fusing and forming myotubes **c** while A6 nuclei remain undifferentiated outward MyHC positive myofibers **a**; nuclei are counterstained with DAPI (blue) **b, d**. The inserts are enlarged view of the dashed area, highlighting H2 enrolment into myotube formation. Scale bar values: **a, c** = 25 μm; inserts = 10μm. **c** Number of LacZ positive nuclei of A6 cells and cell clone in myotubes. Error bars indicate standard deviation. ***P < 0.005

To determine whether the H2 cell clone retained the mab phenotype under normal growth conditions, we assessed morphology, cell doubling time, intracellular ROS levels, expression patterns of certain stemness genes, and differentiation capabilities. In general, there were no differences between mabs and the H2 cell clone among the examined parameters (Supplementary Figs. S2A-C and Fig. 3a); the exception was the myogenic differentiation of the H2 cell clone, which demonstrated an improved ability to form myotubes in co-culture with the C2C12 mouse myoblast cell line (Fig. 3b). To evaluate the myogenic capabilities of selected and unselected mabs, lentiviral infection was performed to label the cells with nuclear β−galactosidase (nLacZ)^26,30^, and the effective participation of the mab nuclei in myotube formation was evaluated, revealing a remarkable increase in H2 myogenic activity compared with that of its A6 counterpart (Fig. 3c).

### Oxidative stress does not affect H2 clone viability, proliferation and migration capacity

**Fig. 4:**
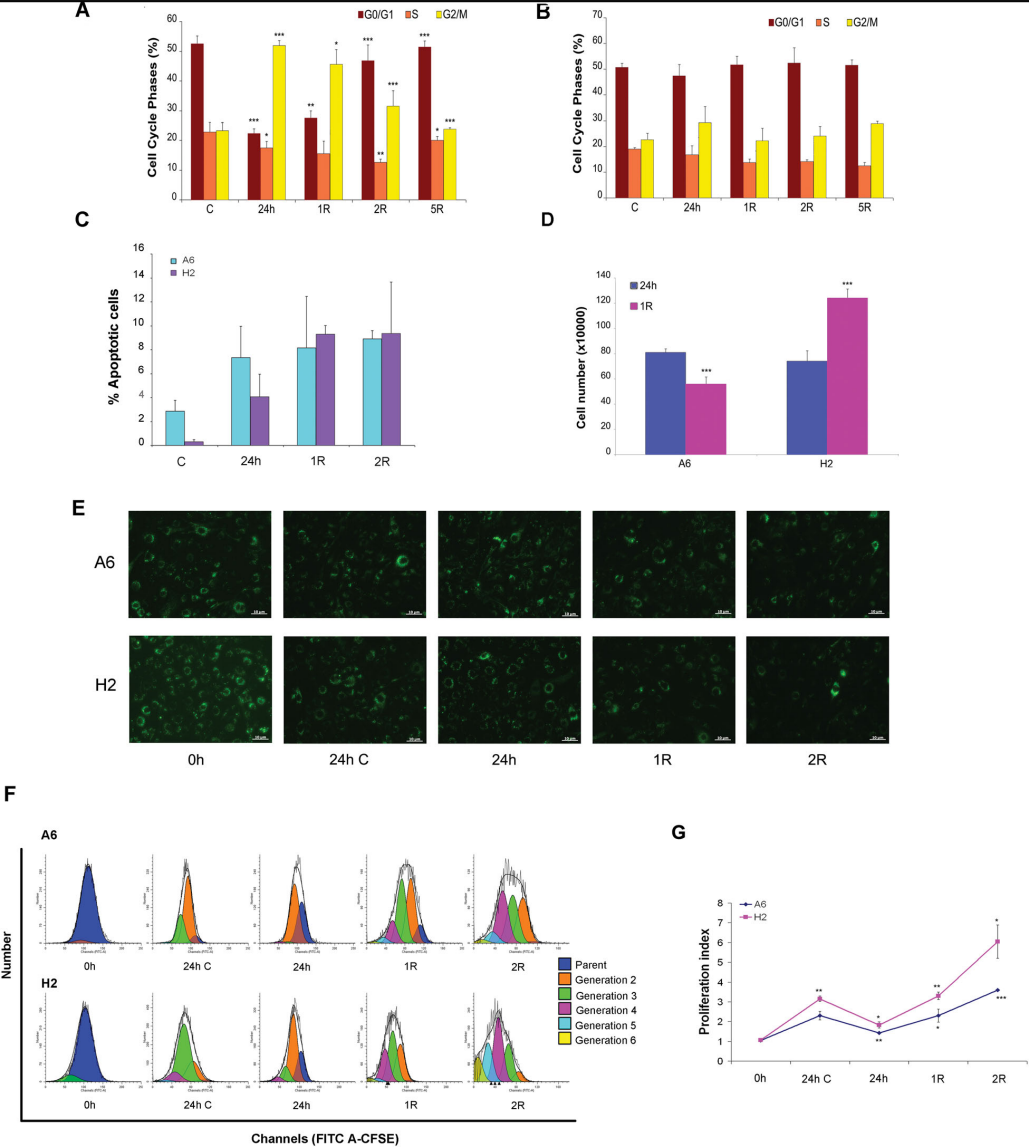
A second oxidative stress does not arrest H2 cells in G_2_/M cell cycle phase **a- b** Cell cycle phase distribution after 200 *μ*M H_2_O_2_ treatment and recovery time. Histograms of cell cycle phase distribution of A6 cells **a** and cell clone **b**: control cells 'C', treated cells (200 *μ*M H_2_O_2_ 24 h) and after 1, 2, 5 days of recovery time are represented. Error bars indicate standard deviation. *P < 0.5, **P < 0.05, ***P < 0.005 vs control group or H_2_O_2_ treated group. **c** Summary of the apoptosis data in histogram form (Annexin V positive+Annexin V and Sytox green double positive cells). **d** Cell number of both A6 cells and cell clone after oxidative stress. Total cell number of A6 cells and cell clone after 24 h of 200 μM H_2_O_2_, and the same at 1 day of recovery period (*R*), Error bars indicate standard deviation. ***P < 0.005. Histograms **a-d ** are representative of three independent experiments. ** e-g** H2 and A6 cell proliferation monitored using CFSE labeling. **e** Representative fluorescence images of CFSE dye labeled A6 and H2 cells incubated with or without 200 µM of H_2_O_2_ and observed at the indicated time point. Showed are typical photographs with 20X magnification. Scale bar is 10 µm. **f** Cells were harvested at different time point and proliferation was analyzed by FACS. Representative analyses from three separate experiments are shown. **g** Proliferation index are reported. Results are expressed as mean of three independent experiments. Error bars indicate standard deviation. *P < 0.1, **P < 0.01, ***P < 0.001 vs 0 h

The sorted cell clone exhibited a behavioural alteration in the presence of an oxidative environment. The cell clone and mabs were subjected to severe oxidative stress using 200 μM H_2_O_2_ for 24 h; this concentration may be similar to that encountered in vivo in chronically inflamed tissues. According to a cell cycle analysis, this lower H_2_O_2_ dose arrested mabs in G_2_/M phase (Fig. 4a) during oxidative stress and recovery, as did the higher dose (400 μM) (see Fig. 2b **)**. In contrast, the cell clone was not arrested in G_2_/M phase, resulting in further analyses (Fig. 4b). We next evaluated the effects of H_2_O_2_ on the viability of both A6 and H2 cells by analysing the cell percentages in different stages of apoptosis using flow cytometry after performing Annexin V-PE/SYTOX Green double staining, which distinguishes between different stages of apoptotic cell death. After H_2_O_2_ treatment, both cell lines showed a marked increase in apoptosis, although the apoptosis levels were higher in A6 than H2 cells (Fig. 4c), confirming the superior resistance of H2 cells to oxidative stress. Then, we characterized the growth capacity of this cell clone, which was doubled compared to the total cell number on day one of recovery after stress induction (Fig. 4d). Conversely, the total number of mabs was reduced, suggesting that mabs and the cell clone behaved differently after exposure to oxidative stress. The proliferative responses of both mabs and H2 cells were also observed after CFSE labelling (Fig. 4e) and quantitatively evaluated using a CFSE-based cytometry assay. After different time points, cell proliferation was assessed by processing the degree of reduction in green fluorescence intensity using ModFit LT^TM^ software (Fig. 4f). As shown in Fig. 4g, H2 cells had a higher proliferation index than A6 cells under basal growth conditions. A significant reduction in this index was observed 24 h after treatment with 200 μM H_2_O_2_. Despite this reduction, H2 cells exhibited a proliferation index higher than that of A6 cells one day after H_2_O_2_ removal. Both H2 and A6 cells demonstrated proliferative capacities comparable to pre-stressed conditions. The H2 proliferative index was doubled after 2 days of recovery compared with that of A6 cells.

**Fig. 5:**
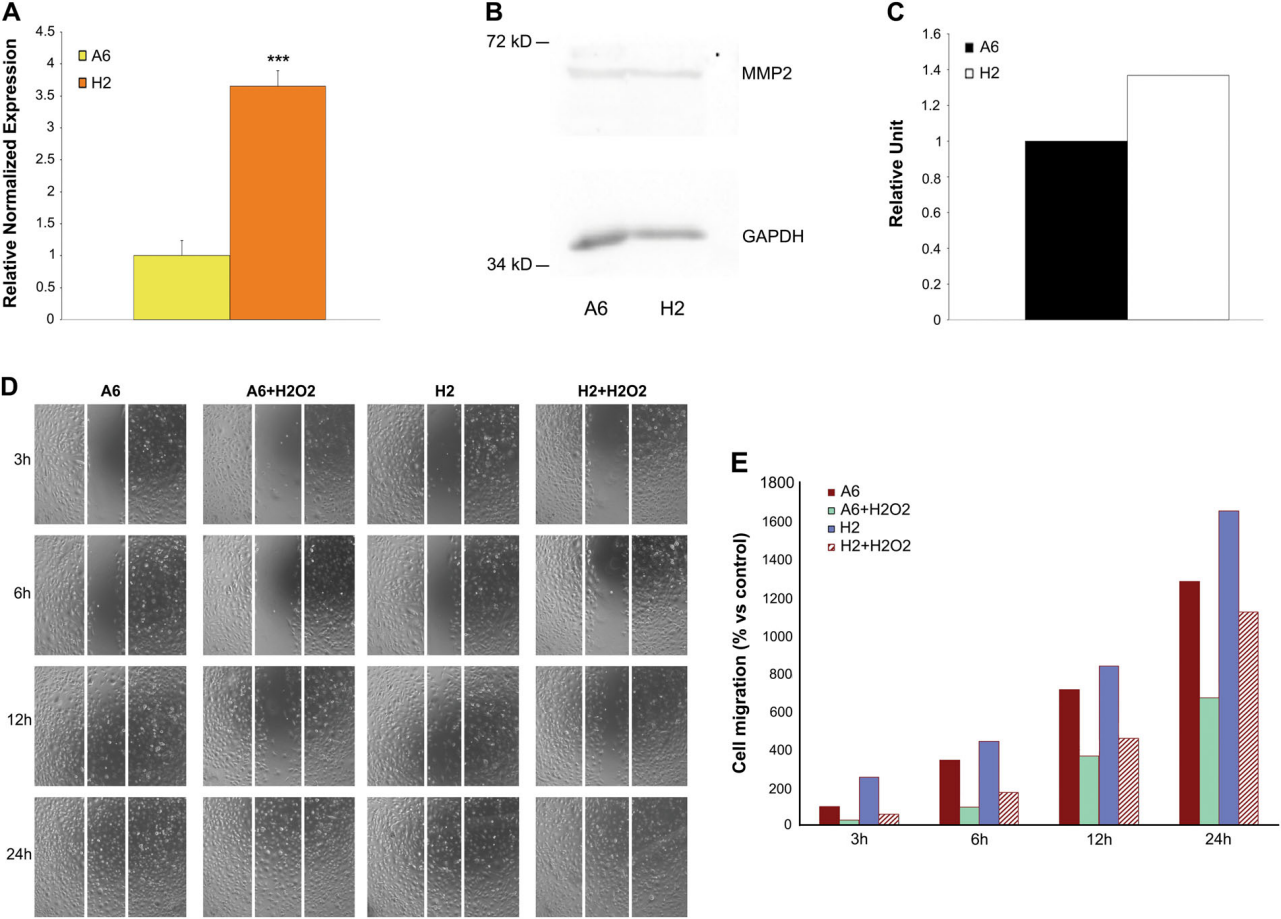
Different amount of MMP2 in cell clone and A6 cells **a** qRT-PCR analysis of MMP2 mRNA in A6 cells and cell clone. Relative quantities of mRNA were first normalized to GAPDH and actin genes, and then A6 samples were arbitrarily set to a value of 1 (n = 3). Data are expressed as mean ± Standard Error of the Mean. ***P < 0.001. **b, c** Western blot assay and histogram of MMP2 in untreated A6 cells and cell clone. The levels of MMP2 were valuated after quantification of immunoreactive bands, obtained by immunoblot assay, by Quantity one software. **d-e** Different migration capability of A6 cells vs H2 cells. Scratch test of A6 cells and cell clone under normal growth conditions and after 200 μM H_2_O_2_ treatment for 24 h ' + H_2_O_2_'. **d** Both cell types were plated in 6–well plates and subjected to wound healing assays and immediately subjected to 200 μM H_2_O_2_ treatment for 24 h. Results are a representative experiment from at least three independently performed experiments with similar results. **e** The number of migrated cells was recorded and the data of three independent experiments are expressed as means. A6 samples were arbitrarily set to a value of 100

Another fundamental stem cell function in transplantation applications is migration capacity; therefore, we evaluated the H2 cell clone for its expression of matrix metalloproteinase 2 (MMP2), which is crucial for migration^31^, to define H2 migratory capacity compared with that of unselected mabs. Real-time PCR and western blot analyses revealed increased MMP2 expression in the H2 cell clone (Fig. 5a–c). To determine whether this increased MMP2 expression was related to enhanced migration capability, we performed a scratch test that revealed effective, increased and rapid cell clone migration under normal growth conditions compared with that of mabs (Fig. 5d, e). We then simulated an in vivo scenario in an in vitro assay and repeated the scratch test in the presence of 200 µM H_2_O_2_ (Fig. 5d, e). The H2 cell clone maintained more rapid migration than treated mabs during the first 24 h of continuous oxidative stress. Based on these results, the H2 cell clone subpopulation was more resistant to an oxidative environment.

### In vivo transplantation of H_2_O_2_-preconditioned mabs

**Fig. 6:**
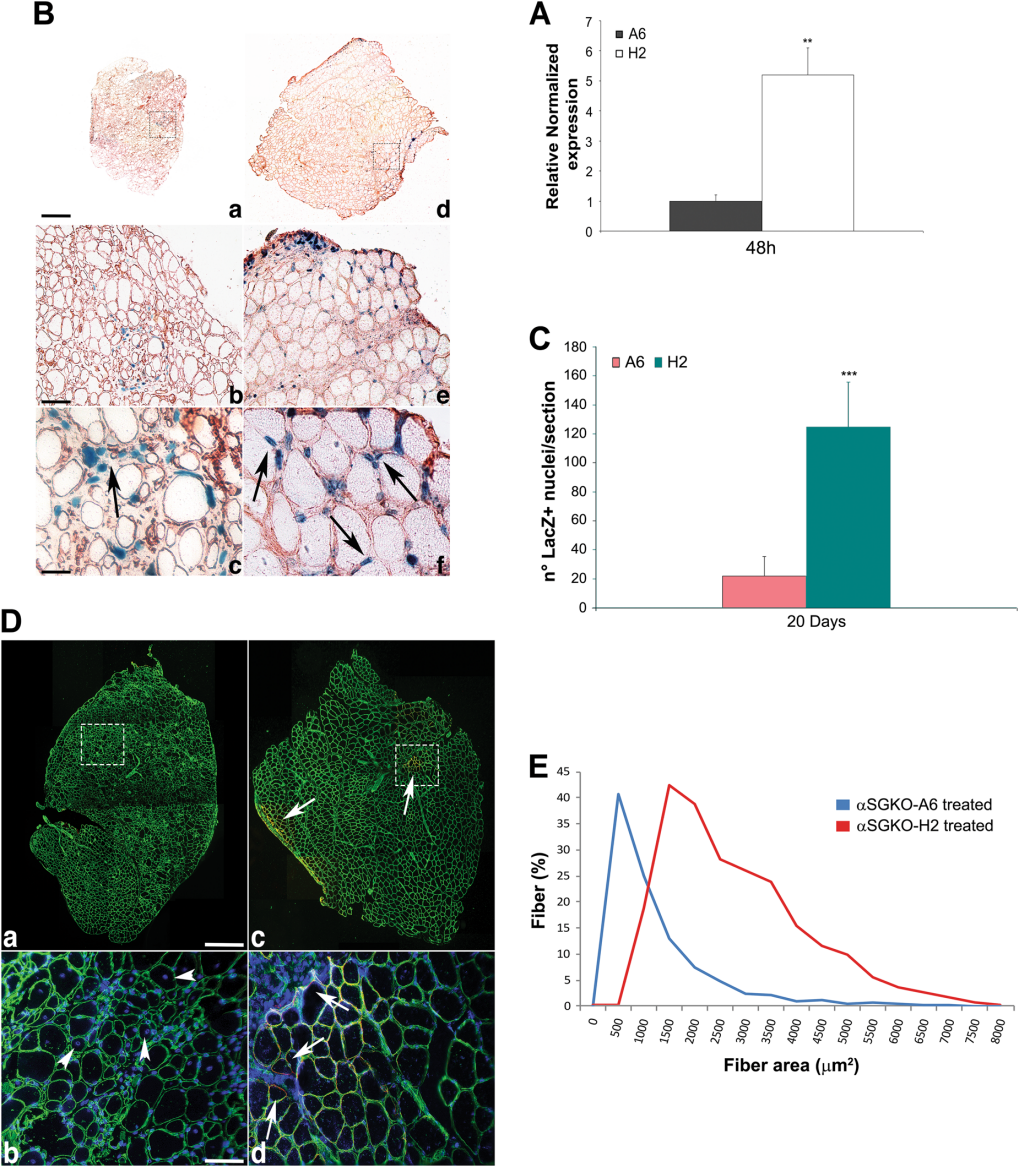
H2 clone transplanted into mouse immunocompromised dystrophic model is more resistant and have a higher integration capability **a** LacZ qRT-PCR of A6 cells and cell clone. RNA was isolated from the treated TA 48 h after mabs intramuscular injection. **b** Immunohistochemistry against laminin (brown) and X-Gal staining (blue) on TA section showing A6 cells **a-c** and H2 cell clone **d-f**, modified for nLacZ and implanted intramuscularly in mouse dystrophic model, after 20 days from injection. Dotted boxes in a and d indicate areas of enlarged view (**b, e**) revealing LacZ positive nuclei into host TA, showing in e integrated LacZ positive nuclei inside regenerating host myofibers. The images in a and d are collage resulting to obtain whole TA section images. **a, d** Magnification 10X, **b, e** magnification 20X, **c, f** magnification 40X. Scale bars: **a** 300 μm; **b**) 40 μm; c 20 μm. **c** Number of LacZ positive nuclei of A6 cells and cell clone injected in the muscle. The data are representative from three independently experiments. The error bars indicate standard deviation. **P < 0.05, ***P < 0.005. **d** Muscle recovery upon 40 days H2 treatment. **a-d** Collage resulting images from TA section immunofluorescence against Laminin (green) and αSarcoglycan (αSG) (red) revealing morphology and overall αSG expression recovery areas (arrows in b) upon H2 intramuscular injection **c, d** comparing with A6 infusion **a, b.** **b, d** Enlarged view of dashed boxes showing still small, centre-nucleated degenerating myofibers (arrowheads) after A6 graft **b** and αSG expression (arrows) and recovered morphology - myofibers with uniform size—upon cell clone injection **d** nuclei were labeled by DAPI (blue). **e** Fibres Cross Sectional Analysis (CSA) highlights the muscle recovery due to H2 effect on αSGKO dystrophic muscle, presenting the disappearance of small (under 500 μm^2^) degenerating muscle fibres

To verify the improved survival and migration capacity of the H2 cell clone, in vivo experiments were performed via the intramuscular injection of cells into an immunocompromised, dystrophic mouse model: SCID/alpha sarcoglycan null mice.^32^ H2 clone cells and unselected wild-type mabs were modified to express nLacZ, and 5 × 10^5^ cells were implanted into the tibialis anterior (TA) muscles of the same mice. The muscles were then collected for real-time PCR (Fig. 6a) and immunohistochemical analysis (Figs. 6b, c) 48 h and 20 days, respectively, after injection. RNA was isolated from the treated TAs, and real-time PCR revealed a remarkable increase in LacZ expression related to the numbers of surviving and engrafted mabs upon implantation (Fig. 6a). β-galactosidase immunostaining also revealed notably high levels of LacZ-positive nuclei in the muscles injected with the H2 cell clone (Fig. 6b d, e) compared with that in the contralateral TA muscles treated with unselected mabs (Fig. 6b a, b, Supplementary Fig. S3). Moreover, high magnification revealed greater spreading within the injected TAs and remarkable integration of H2 cell clone nuclei into regenerating myofibres labelled by laminin immunostaining, reflecting peripheral LacZ nuclei inside host muscle fibres (arrows in Supplementary Figs. S3 and arrows in Figs. 6b, f). We speculate that the higher numbers of lacZ-positive cells observed when H2 cell clones were injected into the TA primarily reflects cell survival; unsorted mabs injected into the dystrophic environment were less resistant and had a lower survival rate (Fig. 6c). Moreover, mice treated with intramuscular H2 cells exhibited a notable ameliorated muscle morphology characterized by more uniform myofibre size and the absence of inflammatory infiltration and degenerating-regenerating areas marked by small fibres, which are clear signs of dystrophy pathology (Fig. 6d). Further supporting this morphofunctional muscle recovery, cross sectional area (CSA) analysis of myofibres clearly revealed the amelioration of muscle fibres upon H2 cell injection, presenting uniform dispersion of fibre size within the recovered muscle; this was also reflected in the recovery of alpha sarcoglycan expression in mice treated with H2 cells (Fig. 6e).

## Discussion

The current study reports that a mouse mesoangioblast stem cell line, A6, is a heterogeneous population. This finding, together with the ability to select clones exhibiting particular behaviours after severe oxidative stress, may have important implications. The results obtained in vitro and in vivo suggest that the use of selected cells demonstrating improvements in specific activities is preferable to using the entire population when addressing issues related to tissue repair. Intra-heterogeneity is an important aspect of stem cell biology, as reflected in human mesenchymal stem cells, which are highly complex at the biochemical level.^33^ In some cases, certain clones derived from a stem cell population have more abundant transcripts than the parental population, and vice versa. Clonal and microarray analyses have shown that cardiac progenitors expressing Mesp1 are distinct populations, most likely due to their regional segregation during early gastrulation.^34^ Here, we isolated a subpopulation from mesoangioblast vessel-associated progenitor cells derived from the mouse embryonic dorsal aorta at stage E9.5.^22,25,35^ The selected subpopulation demonstrated excellent survival following severe oxidative stress for 24 h, while cell death occurred for the majority of treated mabs. Published in vitro results for human mesenchymal stem cells have revealed premature cell senescence upon H_2_O_2_ treatment^19–21^ and permanent growth blockage.^36^ Pomduck and colleagues^37^ improved mesenchymal stem cell resistance to oxidative stress by generating cells with specific protein overexpression profiles. Here, we proposed a selection step to isolate oxidative stress-resistant cells from a heterogeneous population and demonstrated its major advantages. Our results provide reliable evidence supporting the idea that selected cells—or the H2 cell clone—resist cell cycle arrest and are more proliferative. Moreover, the H2 clone migrates faster than mabs in scratch assays both under physiological growth conditions and oxidative stress, based on the observed increased MMP2 expression in these cells, and possess a greater capacity to differentiate. More importantly, H2 cells transplanted into a mouse model of muscular dystrophy were able to considerably ameliorate muscle morphology and restore alpha sarcoglycan expression.

One lingering question is whether surviving cells became resistant due to the negative effects of oxidative stress or due to alterations that occur prior to stress. A study of mesenchymal stem cells demonstrated that specific H_2_O_2_ concentrations induce cell differentiation via the upregulation of Notch signalling, which promotes cell differentiation upon oxidative induction.^38^ In our case, oxidative stress triggered this change/resistance in only a few cells, specifically a subpopulation of mabs, indicating the heterogeneous nature of the starting mab population. Thus, it is possible to promote the cell survival, migration and differentiation capacity of a subpopulation of cells by preconditioning stem cells with an oxidative stress signal, subsequently leaving unaltered or even ameliorating the original capabilities of the preconditioned cells.

In conclusion, our findings highlight the potential of stem cell clone isolation as a therapeutic modality for use in regenerative medicine.

## Materials and methods

### Cell culture and treatment

Mabs were grown on type I collagen treated plates in DMEM (Life Technologies, Carlsbad, CA, USA) supplemented with 10% fetal bovine serum and 1% antibiotic and antimycotic (Life Technologies) in a humidified 5% CO_2_ atmosphere at 37 °C. To induce oxidative stress, cells were grown in medium with H_2_O_2_ (Sigma, St. Louis, MO, USA) at different concentrations. Mabs were treated with H_2_O_2_ only after reaching the confluence of 80%. We used SB 202190 (10 µM for 30 min) (Sigma) as p38 inhibitor.

### Trypan blue dye exclusion

To measure viability, cells were stained with trypan blue (Sigma) dye and then counted as described elsewhere.^39^

### Cytofluorimetric analysis

For cell cycle analysis cells were collected and washed with phosphate buffered saline (PBS) (Life Technologies). Pellets were treated with 0.1% C_6_H_5_Na_3_O_7_, 0.1% NP-40, 50 μg/ml propidium iodide (Life Technologies) and 0.06 mg/ml RNase A (Sigma) for 45 min at RT in the dark. The cells were acquired on a BD FACSCanto (BD Biosciences, Franklin Lakes, NJ, USA) using FACSDiva Software. The analysis was performed using Flowing software 1.6.0.

To determine the intracellular ROS content, cells were collected, washed with PBS and incubated with 2′,7′-dichlorfluorescein-diacetate (Sigma) 5 µM for 30 min at 37 °C. The pellet that had been previously washed with PBS was resuspended again in PBS. Cells were acquires on a BD FACSCanto and analyzed using Flowing software 1.6.0.

### Preparation of protein extracts

Protein extracts were prepared as previously described.^31^ Briefly PBS-washed cells were resuspended in lysis buffer (20 mM Hepes, pH 7.9; 0.4 M NaCl; 0.2 mM EDTA; 10% glycerol; 0.5 mM dithiothreitol; 0.5 mM PMSF; 1X protease/phosphatase inhibitors, Sigma) and lysed by three cycles of freezing (liquid nitrogen) and thawing (37 °C water bath). Cell debris was removed by centrifugation at 17,000 rpm for 30 min at 4 °C.

The extract protein concentrations were determined by the Bradford microassay method (Sigma) using bovine serum albumin as a standard.

### Western blot (immunoblot) analysis

Whole-cell protein extracts were subjected to a 10% SDS-PAGE and transferred to an ECL-Hybond membrane (GE Healthcare Life Sciences, Little Chalfont, UK) using a mini-electroblot (Biorad, Hercules, CA, USA). After blocking for 2 h in 5% nonfat dry milk in TBST buffer (10 mM Tris-HCl, pH 7.5, 150 mM NaCl, 0.2% Tween 20) the membranes were incubated overnight with mouse polyclonal anti-actin (1:1500 dilution, Santa Cruz, Dallas, TX, USA), anti-GAPDH (1:500 dilution Santa Cruz), mouse monoclonal anti-p-p38 (1:200 dilution, Santa Cruz), anti MMP2 (1:1000 dilution, Genetex, Irvine, CA, USA). Peroxidase conjugated anti-rabbit, anti-mouse and anti-rat (1:5000 dilution; Promega, Fitchburg, WI, USA) were used as secondary antibodies. Images are acquired with the Versa-Doc Imaging System (Bio-Rad) and analyzed with the Quantity One software (Biorad).

### Adipocyte differentiation assay

Cells were treated with 10 ng/ml of dexamethasone (Sigma) in complete medium for 5 days and then analyzed for adipocyte morphology. Cells were washed with PBS, and after were fixed with formaldehyde 10% (Sigma) for 90 min. Cells were washed three times with PBS and were then incubated with oil red O (Sigma) for 30 min. After cells are washed with PBS and observed.

### Scratch (wound) assays

Scratch assays was performed as previously described.^31^ Confluent monolayer of mabs and cell clone was wounded by using a 200 µl pipette tip. Cells were washed with DMEM (Life Technologies) (without serum and antibiotics), to remove any detached cells and photographs of the wound were taken. Treatment with H_2_O_2_ 200 µM (Sigma) was added after wound creation. After 3, 6, 12 and 24 h the cells were photographed. All images were made at RT in the medium in which the cells were grown, using a contrast phase microscope (Leica DMIL LED, Wetzlar, DE) with a 4X (HIPLAN NA. 0.10, Leica) objective, magnification 40X and a camera Cool Snap ProColor (Media Cybernetics, Rockville, MD, USA). Four representative extents of cell migration near the wound areas were photographed.

### Quantitaive RT-PCR (qRT-PCR)

Total RNA was extracts with an RNeasy Mini Kit (Qiagen, Hiden, DE), according to the manufacturer’s instructions. qRT-PCR was performed on MMP2. GAPDH and actin were used as internal controls. All reactions were performed by using the i-Script One Step RT-PCR Sybr Green Kit (BioRad) according to the manufacturer’s instructions. The real time RT-PCR primers are summarized in Table 1. qRT-PCR for MMP2 was performed in a total of 25 µl from a mixture containing 0.5 µl of Taq Polymerase with Sybr green mix, 12.5 µl of 2X Buffer, 4.5 µl of forward primer (900 nM), 4.5 µl of reverse primer (900 nM), 1000 ng of RNA for MMP2 gene. Reactions were run in BioRad CFX-96 detector system under the following conditions: 40 cycles of 95 °C for 10 s, 60 °C for 30 s, after pre-incubation at 95 °C for 5 s. All reactions were performed in triplicate. The specificity of the amplification reactions was confirmed by melting-curve analysis. The fold changes in the mRNA expression level of MMP2 in the two cell lines and control sample were compared using the DDCq method (BioRad CFX Manager Software).

Real time RT-PCR for in vivo mabs injection was performed as described.^40^ Briefly, total RNA was extracted by liquid nitrogen homogenization with TRIzol reagent (Life Technologies) following the manual instruction. The obtained RNA was cleaned and DNase treated with RNeasy Mini Kit (Quiagen). Single-stranded cDNA was synthesized with SuperScript First-Strand RT-PCR system (Life Technologies), according to the protocol supplied by the manufacturer. Real time PCR was performed using SYBR GREEN PCR Master Mix (PE Applied Biosystems Foster City, CA, USA) according to the supplied method by intron-spanning primers:

### Sequence of qRT-PCR primers

OligoSequence

GAPDH N5′- ATG TCG TGG AGT CTA CTG GTG T -3′

GAPDH R5′- ATG AGC CCT TCC ACA ATG CCA AAG T -3′

ACTIN N5′- CTG TAT TCC CCT CCA TCG TGG -3′

ACTIN R5′- TCT TGC TCT GGG CCT CGT CA -3′

MMP2 N5′- CAC ATA CAG GAT CAT TGG TTA CAC -3′

MMP2 R5′- ACA GGA AGG GGA ACT TGC AGT A -3′

LacZ N5′-ACT ATC CCG ACC GCC TTA CT -3′

LacZ R5′-TAG CGG CTGA TGT TGA ACT G -3′

### Flow cytometry analysis of cell apoptosis

Apoptosis was measured with Annexin V/PE apoptosis detection kit (BioVison, San Francisco, CA, USA) according to manufacturer’s instructions. Briefly, cells were treated with 200 μM H_2_O_2_ for 24 h, harvested and resuspended in binding buffer with the addition of Annexin V-PE and sytox green. Cells were collected and analyzed by cytometry.

### Proliferation assay

A6 and H2 cells were labeled with CFSE dye using the CFSE Cell Division Tracker Kit (Biolegend, San Diego, CA, USA) according to manufacturer’s instructions. This nontoxic cell-permeant fluorescein dye attaches to cytoplasmic components of cells, resulting in uniform brightness. On cell division, the dye is distributed equally (and thus diluted) between daughter cells. Treatment with H_2_O_2_ 200 µM (Sigma) was added after CFSE labeled and at different time point cells were photographed. All images were obtained using a Zeiss Observer D1 inverted microscope (Zeiss, Oberkochen, Germany) equipped with a Zeiss AxioCam MRm and Axiovision Software (Zeiss). Magnification 20X. For FACS analysis cells were harvested at different time point and analyzed by BD FACSCanto. FITC fluorescence intensities were further analyzed mathematically by ModFit LT™ 3.3 software (Verity Software House Inc., Topsham, ME) to calculate the proliferation index.

### Mesoangioblast trasnsduction with lentiviral vectors

Third-generation lentiviral vectors encoding nuclear β-Galactosidase were employed for mabs transduction, mabs expressing nuclear LacZ (Mabs-nLacZ) were cultured and analyzed in vitro by myogenic fusion and differentiation, and in vivo by intramuscular injection.^26,30^

### Animals and treatments

Three-months-old SCID (Severe Combined Immune Deficiency) Alpha Sarcoglycan (α-SG) null mice were used for intramuscular injection.^31^ Briefly mice were anesthetized with an intramuscular injection of physiologic saline buffer (10 ml/kg) containing ketamine (5 mg/ml) (Bayer, Leverkusen, DE) and xylazine (1 mg/ml) (Bayer). For intramuscular cell delivery, approximately 5 × 10^5^ Mabs-nLacZ were injected into the TA via a 0.20 mm diameter needle inserted along the cranio-caudal axis TA. Mice were sacrificed at different time points for molecular and morphological analysis.

### Immunohistochemistry

The tissue samples were embedded in O.C.T. (BioOptica, Milano, IT**)** and flash-frozen in liquid-N_2_ for 15 sec. Sections were cut at a thickness of 8 μM using a Leika cryostat. The obtained sections were hydrated with PBS and stained with X-Gal (Thermofisher, Waltham, MA, USA**)** to reveal β-galactosidase positive cells as described;^26,30^ briefly sections were washed with PBS (two wash, 5 min each) and incubated 24 h at 37 °C with 'X-Gal working solution'. X-Gal working solution is composed of 'X-gal stock solution' (X-Gal 40 mg/ml in *N*,*N*-dimethyl formamide stored at −20 °C and light protected) diluted 40X in the 'X-Gal dilution buffer' (Potassium Ferricyanide Crystalline 5 mM, Potassium Ferricyanide Trihydrate 5 mM, Magnesium Chloride 2 mM in PBS, light protected and stored at 4 °C). The sections were then incubated with primary antibody (Rabbit anti-Laminin from SIGMA at a 1:100 final concentration) diluted with blocking buffer (PBS with 0.2% Triton X-100 and 20% heat—inactivated goat serum) for 20 min at room temperature (RT). Sections were washed with washing solution (PBS with 0.2% Triton X-100 and 1% Bovine Serum Albumine) and then incubated with secondary antibody (HRP—conjugated goat anti-rabbit from Chemicon, diluted 1:500 in 20% goat serum). The immune reaction was developed using 3-amino-9 ethylcarbazole substrate (AEC, Sigma). Or for H&E staining, sections were washed with PBS (two wash, 5–10 min each), counterstained with aqueous Eosin (Sigma) and then covered directly with aqueous mounting medium, Aqua Poly/Mount (Polysciences, Inc., Warrington, PA, USA) The LacZ positive nuclei were counted for three different nonadjacent transverse sections from the largest TA portion for 3 mice per experimental group.

### Immunofluorescence

Immunofluorescence was performed according with previous publication^41^. Briefly, the cell cultures were fixed in 2% paraformaldehyde (Sigma) for 15 min at 4 °C, then washed with wash buffer (PBS with 0.2% Triton X-100) and incubated with primary antibodies diluted with blocking buffer (PBS with 0.2% Triton X-100 and 20% heat-inactivated goat serum) for 1 hr at RT. The primary antibodies used for cell culture staining were mouse anti Myosin Heavy Chain (MF20) (Developmental Studies Hybridoma Bank, Iowa city, IA) at 1:2 dilution and rabbit anti—lacZ (Cappel) diluted 1:100. For histological section, collected TA were treated as described previously for sectioning then sections were rehydrated in PBS and soaked in blocking buffer (PBS with 0.2% Triton X-100 and 20% heat-inactivated goat serum) for 20 min at RT. Afterwards the sections washed with washing buffer and incubated with primary antibodies diluted with blocking buffer. the primary antibody used were: rabbit anti-Laminin from SIGMA at a 1:100 final concentration and mouse anti-αSarcglycan (Vector, Ad1/20A6) diluted at 1:100. After several washes with washing buffer, sections were incubated with secondary antibodies diluted with blocking buffer for 1 hr at RT. The secondary antibodies used at 1:500 were goat anti-mouse FITC (Chemicon, Waltham, MA, USA) and goat anti-rabbit Alexa488 (Molecular Probes). Specimens were counterstained with DAPI (1:500, Molecular Probes) 10 minutes at RT to detect nuclei, washed several times with wash buffer and photographed with a Nikon Eclipse TE2000 microscope equipped with a CoolSNAP-MYO CCD camera (Photometrix, Kew, AUS) and MetaMorph software (Molecular Devices, Sunnyvale, CA, USA).

### Cross-sectional area analysis

Cryosections stained with anti-laminin antibody were analyzed using an ImageJ macro which automatically detects single fibres within the laminin fluorescent signal. Wrongly detected fibres were manually corrected before to start the CSA measurement. For each sample, the area of more than two thousand single fibres was measured.

### Animal procedures

Experiments on animal were conducted according to the rules of good animal experimentation I.A.C.U.C. n°432 of 12 March 2006 and under ethical approval released on 16/09/2011 from Italian Ministry of Health, protocol #228/2015-PR.

### Statistical analysis

Unpaired two-tailed student’s *t*-test for comparison of two independent groups were performed with GraphPad software (GraphPad Software Inc., La Jolla, CA, USA). Quantitative data are represented as the mean ± standard deviation or SEM of at least three independent experiments.

## Electronic supplementary material

Supplemental Figure 1

Supplemental Figure 2

Supplemental Figure 3

Supplementary Figure Legends
