# Supplementary figures and images for: Oxidative stress preconditioning of mouse perivascular myogenic progenitors selects a subpopulation of cells with a distinct survival advantage in vitro and in vivo
Source: Cell Death Dis. 2018 Jan 3;9(1):1. doi: 10.1038/s41419-017-0012-9 (PMC5849040; doi:10.1038/s41419-017-0012-9)

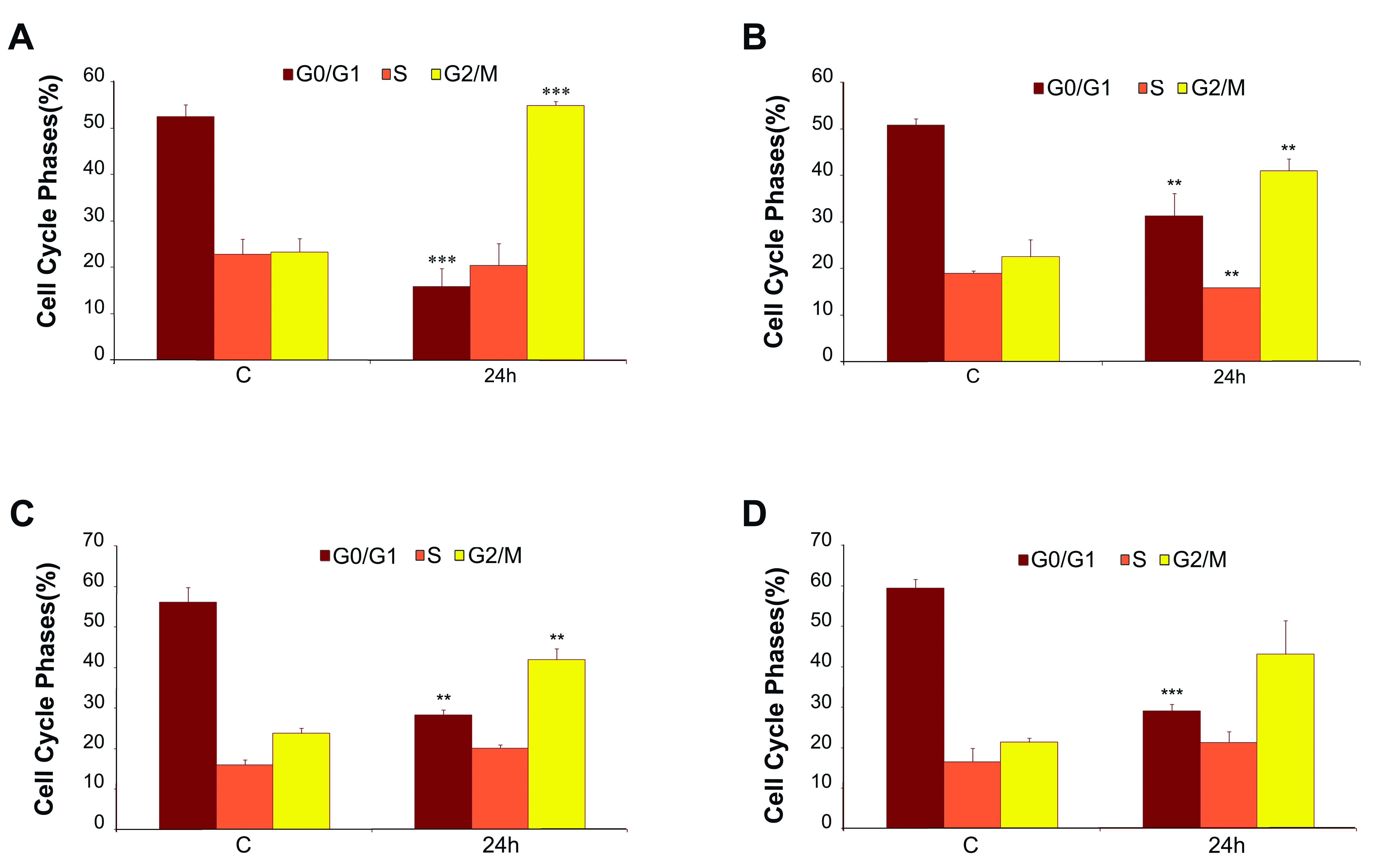

Supplement: Supplementary file 1 — Supplemental Figure 1 [file 41419_2017_12_MOESM1_ESM.jpg]

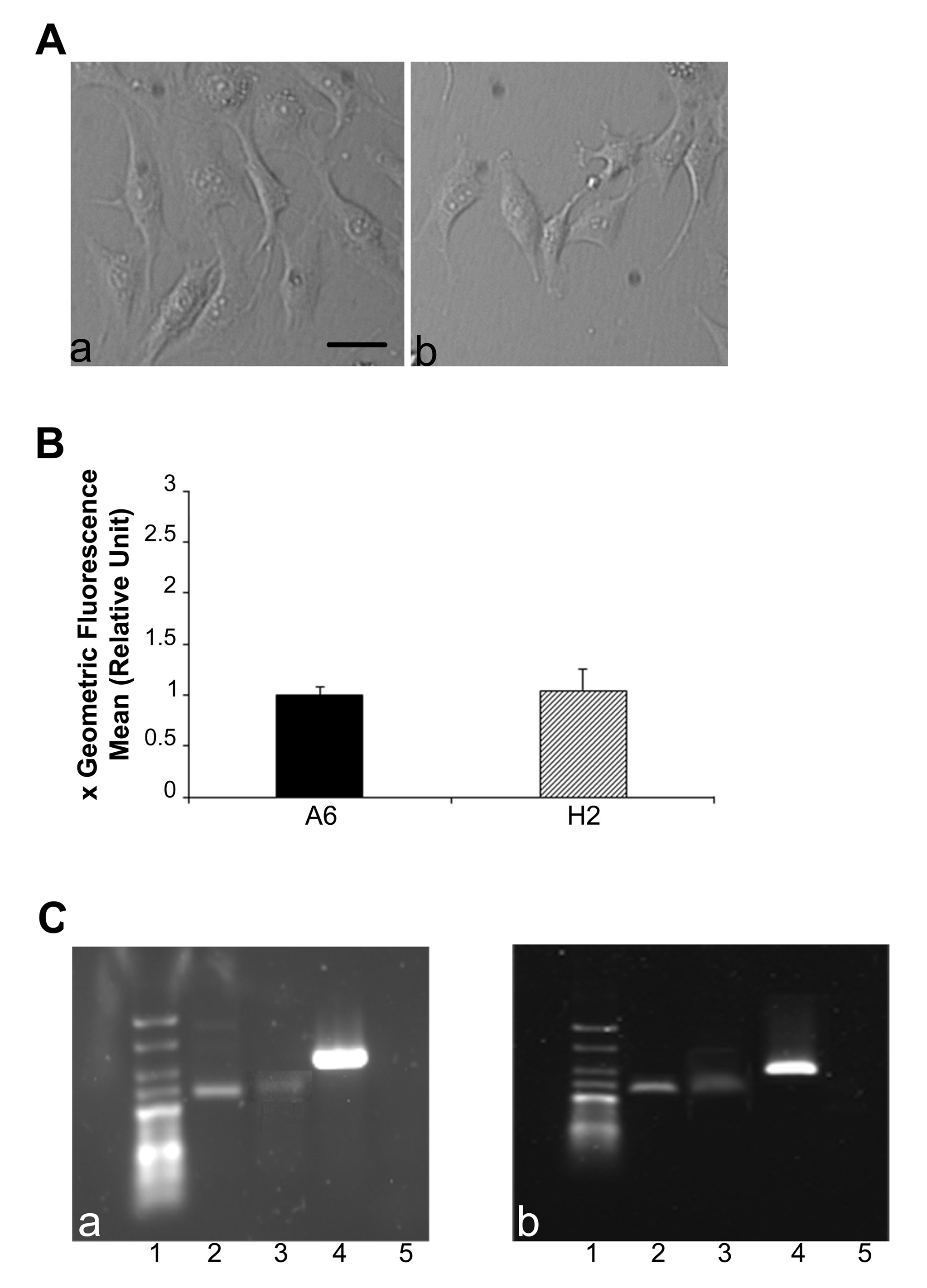

Supplement: Supplementary file 2 — Supplemental Figure 2 [file 41419_2017_12_MOESM2_ESM.jpg]

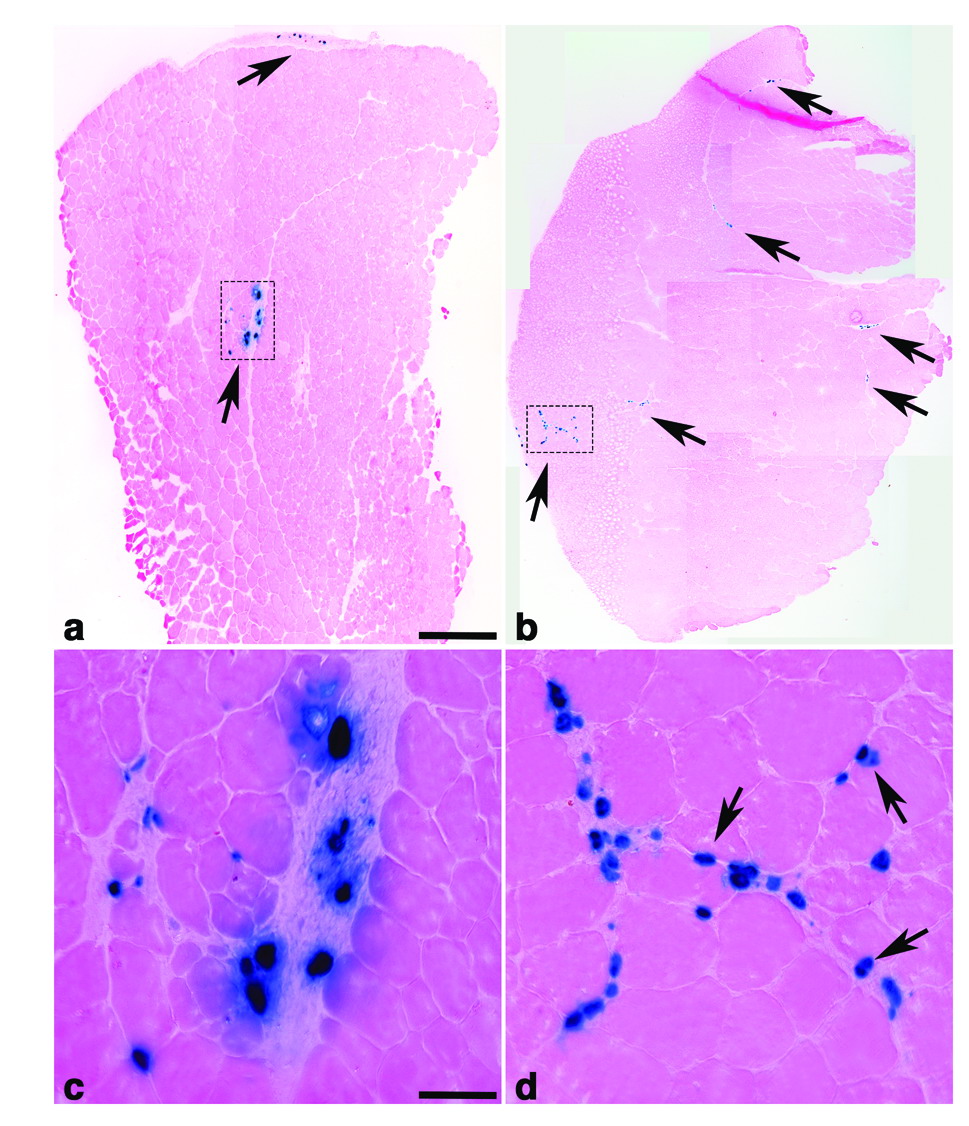

Supplement: Supplementary file 3 — Supplemental Figure 3 [file 41419_2017_12_MOESM3_ESM.jpg]
