# Supplementary material for: Oxidative stress preconditioning of mouse perivascular myogenic progenitors selects a subpopulation of cells with a distinct survival advantage in vitro and in vivo
Source: Cell Death Dis. 2018 Jan 3;9(1):1. doi: 10.1038/s41419-017-0012-9 (PMC5849040; doi:10.1038/s41419-017-0012-9)
Supplement: Supplementary file 4 — Supplementary Figure Legends [file 41419_2017_12_MOESM4_ESM.docx]

**Supplementary Figure Legends**

**Figure S1** Cell cycle phase distribution of A6 cells and isolated cell clones (H2, B9, E3) after oxidative stress. (**A**) control A6 cells “C”, treated A6 cells (400 μM H_2_O_2_ 24h) (**B, C, D**) control “C” and treated “24h” cell clones H2, B9, E3 respectively. Histograms are representative of three independent experiments. Error bars indicate standard deviation. **P<0.05, ***P<0.005.

**Figure S2**

Basal phenotypes of A6 cells and H2 cell clone. Cell doubling time is 16h for both A6 and H2 cells.^42^ (**A**) Same morphology between A6 cells (a) and cell clone (b). Shown are typical photographs from three independent experiments. Magnification 20X. Scale bar is 15 μm. (**B**) ROS content in untreated A6 cells and untreated cell clone. (**C a-b**) Expression profile of some stemness genes by RT-PCR of A6 cells (a) and cell clone (b): markers (lane 1); VE-cadherin (lane 2); Flk1 (lane 3); CD34 (lane 4); Myf5, negative control (lane5).

**Figure S3** β−Gal labeling on HE stained TA sections. A6 cells (a, c) and cell clone (b, d), modified for nLacZ expression, implanted intramuscularly in mouse dystrophic model, after twenty days from injection. Arrows indicate the LacZ nuclei inside the host muscle fibers. The images in a and b are collage resulting to obtain whole section images, c and d enlarged view of dotted box in a and c. Scale bars: a=300 μm; b=20 μm.
